# A biomarker feasibility study in the South East Asia Community Observatory health and demographic surveillance system

**DOI:** 10.1017/gheg.2018.13

**Published:** 2018-08-22

**Authors:** U. Partap, E. H. Young, P. Allotey, M. S. Sandhu, D. D. Reidpath

**Affiliations:** 1 Department of Medicine, University of Cambridge, United Kingdom; 2 Wellcome Sanger Institute, Hinxton, United Kingdom; 3 United Nations University International Institute for Global Health (UNU-IIGH), Kuala Lumpur, Malaysia; 4 Jeffrey Cheah School of Medicine and Health Sciences, Monash University Malaysia, Selangor, Malaysia; 5 South East Asia Community Observatory, Segamat, Malaysia

**Keywords:** South East Asia, health and demographic surveillance, feasibility studies, biological sample collection

## Abstract

**Background:**

Integration of biomarker data with information on health and lifestyle provides a powerful tool to enhance the scientific value of health research. Existing health and demographic surveillance systems (HDSSs) present an opportunity to create novel biodata resources for this purpose, but data and biological sample collection often presents challenges. We outline some of the challenges in developing these resources and present the outcomes of a biomarker feasibility study embedded within the South East Asia Community Observatory (SEACO) HDSS.

**Methods:**

We assessed study-related records to determine the pace of data collection, response from potential participants, and feedback following data and sample collection. Overall and stratified measures of data and sample availability were summarised. Crude prevalence of key risk factors was examined.

**Results:**

Approximately half (49.5%) of invited individuals consented to participate in this study, for a final sample size of 203 (161 adults and 42 children). Women were more likely to consent to participate compared with men, whereas children, young adults and individuals of Malay ethnicity were less likely to consent compared with older individuals or those of any other ethnicity. At least one biological sample (blood from all participants – finger-prick and venous [for serum, plasma and whole blood samples], hair or urine for adults only) was successfully collected from all participants, with blood test data available from over 90% of individuals. Among adults, urine samples were most commonly collected (97.5%), followed by any blood samples (91.9%) and hair samples (83.2%). Cardiometabolic risk factor burden was high (prevalence of elevated HbA1c among adults: 23.8%; of elevated triglycerides among adults: 38.1%; of elevated total cholesterol among children: 19.5%).

**Conclusions:**

In this study, we show that it is feasible to create biodata resources using existing HDSS frameworks, and identify a potentially high burden of cardiometabolic risk factors that requires further evaluation in this population.

## Introduction

There is a need for comprehensive data resources on population health and disease in low- and middle-income countries, where a large proportion of the global burden of morbidity and mortality is located (1, 2). Biomarker data form an essential component of such endeavours, allowing objective assessment of a wide range of disease-related indices, facilitating validation of self-reported information, and allowing for greater statistical power of analyses. Integration of biomarker data with information on health and lifestyle provides a powerful tool to enhance the scientific value of health research.

Large-scale surveys in low- and middle-income populations, such as the Demographic and Health Surveys, have previously included biomarker modules (3). However, these have often been restricted to a narrow range of measures from limited samples, with variable capacity for long-term storage and later analysis (3). Importantly, they are unable to follow up individuals over time. Health and demographic surveillance system (HDSS) sites offer a valuable opportunity for efficient, large-scale collection and analysis of biomarker data. They provide pre-existing infrastructure to facilitate biological sample collection, and the potential to link biomarker data longitudinally to historical and future measures. This linkage allows for a detailed view of disease development across the life course (4).

We undertook a biomarker feasibility study embedded within the South East Asia Community Observatory (SEACO) HDSS, which covers approximately 45 000 individuals in Segamat, Malaysia (5). The SEACO HDSS conducts annual enumeration of individuals, and has also undertaken a population-wide health survey collecting questionnaire data and biophysical measurements, in its catchment area (5). Through this study, we explored the feasibility of building upon the previous survey work conducted by SEACO to include biological sample collection. This feasibility study aimed to recruit approximately 200 individuals aged seven years and above to assess the preparedness of individuals and families to participate, and to establish the procedures for the collection, analysis and storage of biological samples within a predominantly rural community setting. Here, we outline the developments in the procedures and examine the outcomes of this study to determine the potential to create a large-scale biodata resource within the full HDSS population.

## Methods

A detailed profile of the SEACO HDSS, including the HDSS development, structure, and data collections, is presented in a recent publication (5).

### Sampling

Adult (aged 18 years and over) and child (aged 7–17 years) participants for this study were recruited from the SEACO HDSS (5). Stratified random sampling was performed at the household level using data from the most recent enumeration (completed in 2016), aiming to achieve comparable proportions of individuals of Malay, Indian, Chinese and Orang Asli (indigenous) ethnicity. Sampling therefore covered all enumerated households within the SEACO catchment area (approximately 1250 km^2^). SEACO has established strong community links through its community engagement strategy (6), and additional community awareness activities were undertaken to sensitise potential participants prior to this study.

### Data and sample collection

Community-based data and sample collection was undertaken by two field teams between November 2016 and February 2017. Data were recorded on electronic tablets. Informed consent (adults) or informed assent with parental or guardian consent (children) was first obtained; individuals could only participate if they consented to providing all data and samples (Supplementary Methods). Following informed consent, along with questionnaire and biophysical data, capillary blood (via finger prick, for point-of-care glycated haemoglobin [HbA1c] measurement), and venous blood (four tubes from a single blood draw: up to 24 ml from adults, 12 ml from children; for serum, plasma and whole blood samples) were collected from participants. Hair and urine samples were also collected from adult participants. Following data and sample collection, participants were given their body mass index (BMI), blood pressure and point-for-care HbA1c results, and were provided referral to local clinics if these were above pre-determined cut-offs. One session of data and sample collection took approximately 40–50 minutes for adult participants and 30 minutes for children (see Supplementary Methods for further details on sample collection purposes and procedures).

### Measures and statistical analysis

#### Study measures to evaluate scale-up

Literature on suitable measures or assessment frameworks to determine feasibility for population-based observational studies is scarce (7–10). We therefore identified and examined a range of study-related measures to gain a comprehensive picture of the potential for scale-up. This included indicators of efficiency, response from potential participants, feedback from participants, and completeness and quality of collected data and samples.

First, we summarised study operational data to assess operational efficiency and response to the study. This assessment included information on the number of days of data and sample collection; the number and demographic characteristics of households and individuals approached; proportions consenting, declining or absent; reasons for refusal among those declining participation; and post-study feedback among participating individuals. Study pace was calculated as the average number of participants recruited per day. Differences in demographic characteristics between consenting and non-consenting individuals were assessed using Pearson's chi squared tests or Fisher's exact tests (cell counts less than five).

Following this, we examined measures relating to quality and completeness of data and samples. We were particularly interested in measures relating to blood sample collection, availability of blood test data and availability of blood sample aliquots, as indicators of the success of sample collection, analysis and storage. We extracted relevant information from three datasets generated at the end of the study: (i) data recorded on the electronic questionnaire form, (ii) blood test results, and (iii) records of receipt, processing and aliquoting of biological samples at the central research laboratory. All three datasets were cleaned, merged and checked for consistency. The completeness of questionnaire data for each participant was assessed by examining a set of all questions and measurements collected from all participants. The number of participants with any questionnaire data, blood test data, collected samples and samples for storage (plasma, serum, whole blood and remnant cell aliquots, urine aliquots and hair samples) was examined, and differences by sex, ethnicity and obesity status were assessed. The number of participants with complete data and samples was similarly examined.

#### Sociodemographic, lifestyle and risk factor data

Finally, sociodemographic characteristics of study participants and crude prevalence of key lifestyle, biophysical and blood-based risk factors in the population were examined; differences by sex were assessed using Pearson's chi squared or Fisher's exact tests (see Supplementary Methods for list of variables and corresponding definitions).

All data management and analyses were performed using Stata 14 (Statacorp, Texas).

### Ethical approvals

Ethical approval for the study was obtained from the Monash University Human Research Ethics Committee (CF16/471–2016000227), and approval for the receipt and analysis of linked anonymised data at the University of Cambridge was obtained from the University Human Biology Research Ethics Committee (HBREC.2017.04) (Supplementary Methods).

## Results

### Study measures to evaluate scale-up

#### Measures of study recruitment and response

**Fig. 1.**
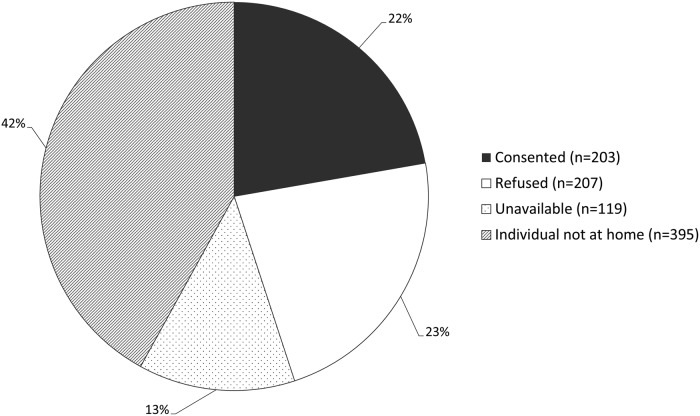
Proportion of individuals (N = 912) in visited houses (N = 289) who consented, refused, were not at home or were unavailable. Unavailable: individuals from visited houses who were found to have moved away (n = 107) or passed away since the most recent enumeration (n = 12). Individual not at home: eligible individuals who were not at home at the time of the visit.

Overall, 203 participants (161 adults, 42 children) were recruited into the biomarker feasibility study, close to half (49.5%) of those responding to an invitation to participate (Figure 1, Table 1). A notable proportion of houses was empty upon approach, either due to the household having moved away (n = 107; 11.7%), or household members not being at home (n = 383; 42.0%) (Figure 1). Recruitment and data and sample collection occurred over 48 working days, with an overall study pace of 4.2 participants per day (Supplementary Figures S1–S2). Among households providing reasons for refusal to participate (63.5%), the most common included disinterest (16.2%) or fear of needles (24.3%) (Supplementary Table S1).

**Table 1.** Summary of individuals living in houses visited by the study team.

	Eligible individuals living in visited houses^1^ (n, %)		Invited^2^ (n, % of eligible individuals)		Consented (n, % of invited)		Refused (n, % of invited)		*P* (consented versus refused)
Adults									
N	516		270	(52.3)	161	(59.6)	109	(40.4)	
Sex									
Male	250	(48.4)	121	(48.4)	59	(48.8)	62	(51.2)	
Female	266	(51.6)	149	(56.0)	102	(68.5)	47	(31.5)	0.001
Age category (years)									
18–29	148	(28.7)	56	(37.8)	27	(48.2)	29	(51.8)	
30–39	93	(18.0)	46	(49.5)	31	(67.4)	15	(32.6)	
40–49	103	(20.0)	52	(50.5)	35	(67.3)	17	(32.7)	
50–59	87	(16.9)	61	(70.1)	36	(59.0)	25	(41.0)	
60+	85	(16.5)	55	(64.7)	32	(58.2)	23	(41.8)	0.239
Ethnicity									
Malay	191	(37.0)	104	(54.5)	43	(41.3)	61	(58.7)	
Indian	145	(28.1)	57	(39.3)	41	(71.9)	16	(28.1)	
Chinese	102	(19.8)	58	(56.9)	37	(63.8)	21	(36.2)	
Bumiputera/Orang Asli	77	(14.9)	50	(64.9)	39	(78.0)	11	(22.0)	
Other	1	(0.2)	1	(100.0)	1	(100.0)	0	(0.0)	<0.001
Children									
N	277		140	(50.5)	42	(30.0)	98	(70.0)	
Sex									
Male	142	(51.3)	73	(51.4)	23	(31.5)	50	(68.5)	
Female	135	(48.7)	67	(49.6)	19	(28.4)	48	(71.6)	0.685
Age category (years)									
7–12	161	(58.1)	77	(47.8)	17	(22.1)	60	(77.9)	
13–17	116	(41.9)	63	(54.3)	25	(39.7)	38	(60.3)	0.024
Ethnicity									
Malay	93	(33.6)	42	(45.2)	8	(19.0)	34	(81.0)	
Indian	81	(29.2)	35	(43.2)	9	(25.7)	26	(74.3)	
Chinese	59	(21.3)	32	(54.2)	12	(37.5)	20	(62.5)	
Bumiputera/Orang Asli	44	(15.9)	31	(70.5)	13	(41.9)	18	(58.1)	0.129
Other	0	(0.0)	0	n/a	0	n/a	0	n/a	

^1^Individuals aged seven years or above and covered in the most recent HDSS enumeration, who had not moved away or passed away since the most recent enumeration.

^2^Eligible individuals who were available to respond to an invitation to participate in the study at the time of visit.

Differences in distributions across categories of response were compared using Pearson's chi squared or Fisher's exact (cell counts < 5) tests.

A greater proportion of women (56%) versus men, individuals aged 50–59 years (70.1%) or 60 years and above (64.7%) versus younger individuals, and those of Orang Asli ethnicity (64.9% among adults, 70.5% among children) versus those of any other ethnicity were available during recruitment (Table 1). Of those available and subsequently invited, women (68.5%, *P* < 0.001) were more likely to consent to participate compared with men, whereas children (30.0%) and young adults (48.2%), and those of Malay ethnicity (adults: 41.3%, *P* < 0.001, children: 19.0%, *P* = 0.129) were less likely to consent, compared with older individuals or those of any other ethnicity (Table 1).

Of 170 (83.7%) participants providing post-study feedback, over 95% agreed with comments relating to a favourable experience, including comfort during questionnaire administration (99.4%), interest in the study results (100.0%), and willingness to encourage others to participate in the study (99.4%) (Supplementary Table S2).

#### Completeness and quality of data and samples

We then examined the availability of data and samples collected from participants. All participants had some available questionnaire information, with most having three or fewer missing variables (Table 2, Supplementary Tables S3-S4). At least one biological sample (capillary blood, venous blood, hair or urine) was collected at the anticipated quantity from all individuals (Table 2, Supplementary Table S5). Over 90% of participants had some blood test data, whilst approximately 70–80% had complete data (Table 2), with no systematic differences in data and sample availability by ethnicity (Supplementary Figures S3–S4).

**Table 2.** Detailed summary of data and sample collection completeness, stratified by sex.

	n (%)											
	Adults						Children					
	Overall		Men		Women		Overall		Boys		Girls	
N	161		59		102		42		23		19	
Questionnaire data												
Any questionnaire data post-cleaning	161	(100.0)	59	(100.0)	102	(100.0)	42	(100.0)	23	(100.0)	19	(100.0)
Complete questionnaire data post-cleaning	13	(8.1)	6	(10.2)	7	(6.9)	12	(28.6)	8	(34.8)	4	(21.1)
Samples for point-of-care analysis												
Capillary (finger-prick) blood sample successfully obtained	161	(100.0)	59	(100.0)	102	(100.0)	42	(100.0)	23	(100.0)	19	(100.0)
Samples for analysis and storage collected at full volume												
Any venous blood sample^1^	143	(88.8)	57	(96.6)	86	(84.3)	41	(97.6)	23	(100.0)	18	(94.7)
All venous blood samples^2^	111	(68.9)	43	(72.9)	68	(66.7)	38	(90.5)	22	(95.7)	16	(84.2)
Any venous blood sample^1^ or hair sample or urine sample	161	(100.0)	59	(100.0)	102	(100.0)						
Any venous blood sample^1^ and hair sample and urine sample	115	(71.4)	34	(57.6)	81	(79.4)						
All venous blood samples^2^ and hair sample and urine sample	91	(56.5)	26	(44.1)	65	(63.7)						
Venous blood samples for analysis and storage collected at full volume												
Plain serum blood sample	141	(87.6)	57	(96.6)	84	(82.4)	39	(92.9)	22	(95.7)	17	(89.5)
EDTA (plasma) blood sample	139	(86.3)	55	(93.2)	84	(82.4)	41	(97.6)	23	(100.0)	18	(94.7)
EDTA (whole blood 1) blood sample	131	(81.4)	52	(88.1)	79	(77.5)	40	(95.2)	23	(100.0)	17	(89.5)
EDTA (whole blood 2) blood sample	115	(71.4)	48	(81.4)	69	(67.6)	40	(95.2)	23	(100.0)	17	(89.5)
Hair and urine samples collected												
Hair sample	134	(83.2)	35	(59.3)	99	(97.1)						
Urine sample (full volume)	157	(97.5)	59	(100.0)	98	(96.1)						
Blood analysis data												
Any blood test data	148	(91.9)	58	(98.3)	90	(88.2)	41	(97.6)	23	(100.0)	18	(94.7)
Complete blood test data	125	(77.6)	53	(89.8)	72	(70.6)	29	(69.0)	16	(69.6)	13	(68.4)

EDTA: ethylene diamine tetra-acetic acid.

^1^At least one of: plain serum or EDTA (plasma) or EDTA (whole blood 1) or EDTA (whole blood 2).

^2^All of: plain serum and EDTA (plasma) and EDTA (whole blood 1) and EDTA (whole blood 2).

Given the potential to obtain detailed biomarker information from blood, the availability and quality of blood samples was of particular interest in this study. A capillary (finger-prick) blood sample was successfully collected from all participants, with successful point-of-care HbA1c measurement in almost all (99.0%) participants (Table 2). At least one venous blood sample of any volume was collected from over 90% of both adult and child participants, with 82.6% of adults and 95.2% of children having all four blood samples collected at any volume (Table 3; Supplementary Tables S6-S7). Notably, obese adults were less likely to have blood samples successfully collected (at least one blood sample at any volume: 100% among non-obese adults versus 79.5% among obese adults, *P* = 0.002) (Supplementary Table S8). Almost all collected blood samples passed as acceptable quality by the research laboratory, for processing, analysis and storage (Table 3; Supplementary Tables S6-S7). At least one storage aliquot was available from all collected and accepted blood samples among children, and over 96.2% of samples among adults (Supplementary Tables S9-S10).

**Table 3.** Summary of venous blood sample collection completeness and quality from adults and children.

	n (%)							
	Any volume		Full volume		Any volume + accepted by laboratory		Full volume + accepted by laboratory	
Adults (N = 161)								
Plain serum blood sample	148	(91.9)	141	(87.6)	145	(90.1)	141	(87.6)
EDTA (plasma) blood sample	142	(88.2)	139	(86.3)	142	(88.2)	139	(86.3)
EDTA (whole blood 1) blood sample	141	(87.6)	131	(81.4)	139	(86.3)	131	(81.4)
EDTA (whole blood 2) blood sample	133	(82.6)	115	(71.4)	132	(82.0)	115	(71.4)
At least one blood sample	148	(91.9)	143	(88.8)	145	(90.1)	143	(88.8)
All blood samples	133	(82.6)	111	(68.9)	132	(82.0)	111	(68.9)
Children (N = 42)								
Plain serum blood sample	41	(97.6)	39	(92.9)	40	(95.2)	39	(92.9)
EDTA (plasma) blood sample	41	(97.6)	41	(97.6)	41	(97.6)	41	(97.6)
EDTA (whole blood 1) blood sample	40	(95.2)	40	(95.2)	40	(95.2)	40	(95.2)
EDTA (whole blood 2) blood sample	40	(95.2)	40	(95.2)	40	(95.2)	40	(95.2)
At least one blood sample	41	(97.6)	41	(97.6)	41	(97.6)	41	(97.6)
All blood samples	40	(95.2)	38	(90.5)	39	(92.9)	38	(90.5)

EDTA: ethylene diamine tetra-acetic acid.

### Sociodemographic, lifestyle and risk factor data

In addition to a notable prevalence of lifestyle and biophysical risk factors, we found a high burden of blood-based cardiometabolic risk factors in this population. Close to one quarter of adults (23.8%) had elevated HbA1c, while 8.2% had elevated total cholesterol, 15.0% had low HDL cholesterol, and 38.1% had elevated triglycerides (Table 4). Risk factor prevalence was similarly high among children: 19.5% had elevated total cholesterol, 14.6% had low HDL cholesterol and 36.6% had elevated triglycerides (Table 4).

**Table 4.** Crude prevalence of selected lifestyle, biophysical and blood-based risk factors in the study population.

	Number and crude prevalence (n, %)													
	Overall		Men		Women		*P*	Overall		Boys		Girls		*P*
N	161		59 (36.7)		102 (63.4)			42		23 (54.8)		19 (45.2)		
Current smoker	24	(14.9)	22	(37.3)	2	(2.0)	<0.001	2	(4.8)	2	(8.7)	0	(0.0)	0.492
Any alcohol consumption in past 12 months	20	(12.4)	14	(23.7)	6	(5.9)	0.001	1	(2.4)	1	(4.4)	0	(0.0)	1.000
Low fruit and vegetable consumption	157	(98.1)	59	(100.0)	98	(97.0)	0.297	38	(92.7)	22	(95.7)	16	(88.9)	0.573
Insufficient physical activity	63	(39.1)	18	(30.5)	45	(44.1)	0.088							
Overweight	97	(60.6)	32	(54.2)	65	(64.4)	0.206	10	(23.8)	5	(21.7)	5	(26.3)	0.729
Obesity	39	(24.4)	10	(17.0)	29	(28.7)	0.095	3	(7.1)	0	(0.0)	3	(15.8)	0.084
Underweight								5	(11.9)	5	(21.7)	0	(0.0)	0.053
Stunting								3	(7.1)	2	(8.7)	1	(5.3)	0.573
Central obesity	100	(62.5)	26	(44.1)	74	(73.3)	<0.001	1	(2.4)	0	(0.0)	1	(5.3)	0.452
Elevated waist to hip ratio	84	(52.5)	34	(57.6)	50	(49.5)	0.321							
Hypertension^1^	71	(44.1)	30	(50.9)	41	(40.2)	0.190	6	(14.3)	5	(21.7)	1	(5.3)	0.197
Elevated HbA1c^2^	38	(23.8)	8	(13.6)	30	(29.7)	0.021	0	(0.0)	0	(0.0)	0	(0.0)	
Anaemia	26	(16.2)	0	(0.0)	26	(25.5)	<0.001	4	(9.5)	0	(0.0)	4	(21.1)	0.035
Elevated total cholesterol^1^	12	(8.2)	5	(8.6)	7	(7.9)	0.870	8	(19.5)	2	(8.7)	6	(33.3)	0.109
Low HDL cholesterol	22	(15.0)	11	(19.0)	11	(12.4)	0.345	6	(14.6)	4	(17.4)	2	(11.1)	0.679
Elevated triglycerides	56	(38.1)	26	(44.8)	30	(33.7)	0.175	15	(36.6)	9	(39.1)	6	(33.3)	0.702

HbA1c: glycated haemoglobin. HDL: high-density lipoprotein.

Classification of all risk factors is described in the Supplementary Methods.

Differences in distributions between men and women or boys and girls were assessed using Pearson's chi squared or Fisher's exact (cell counts < 5) test.

N was reduced due to missing observations for the following measures: (1) Low fruit and vegetable consumption among girls (N = 18); (2) Overweight, obesity, central obesity and elevated waist to hip ratio and elevated HbA1c among women (N = 101); (3) Elevated HbA1c in girls (N = 18); (4) All cholesterol and triglyceride measures among girls (N = 18), men (N = 58) and women (N = 89).

^1^Measures for hypertension and elevated cholesterol prevalence included individuals who reported being told they had elevated blood pressure or cholesterol.

^2^HbA1c as measured at the point of care.

## Discussion

Detailed, objective measures provided by biomarker information are fundamental to comprehensive data resources on population health and disease. In this study, we show the feasibility of biomarker collection within the context of the SEACO HDSS. Approximately half of invited individuals consented to participate in biological sample collection, with favourable participant feedback. Biological samples were collected from all participants. Outcome measures indicated that there was scope to increase study pace, and a need to improve blood sample collection from obese participants, both attainable through appropriate modifications to study design and training. A high prevalence of blood-based cardiometabolic risk factors was observed among both adult and child participants. These results indicate that creation of a large-scale biodata resource is both achievable and valuable in this population, with potential relevance to similar HDSS sites.

We demonstrate here that capitalising on existing HDSS frameworks to undertake biomarker collection is an efficient way to encourage community participation, and to enhance their value as data resources. We undertook biological sample collection by building upon the strong existing infrastructure, data, human and material resources, local knowledge and community and administrative links established by the SEACO HDSS (5). The proportion of consenting versus invited participants observed in this study is comparable to or greater than other large-scale biobank or biomarker collection studies based in high-income countries (11, 12). Participants were willing to provide both capillary and venous blood samples, with successful capillary blood collection for all participating individuals. Blood test data and storage aliquots were available for the majority of participants, indicating the successful establishment of procedures from sample collection to analysis and long-term storage. Data and sample collection took under an hour, and participants providing feedback responded favourably to the study. The community engagement strategy previously established by SEACO provided a mechanism through which individuals could raise and address concerns they had with participation in this study (6). Importantly, we have the capacity to link information obtained in this study with measures from both previous and future HDSS data collections, including later clinical outcomes, which will facilitate the creation of richer datasets that may be explored in future analyses.

Compared with the growing focus on feasibility studies for randomised clinical trials (13–24), literature on operational outcomes of observational feasibility studies remains scarce, and restricted to a limited number of measures, such as the overall proportion of invited individuals ultimately participating (7–10). Few studies have directly assessed measures of sample collection feasibility, with none identified here that specifically examined blood sample collection (7, 25). Here, we identified useful indicators relating to various aspects of study operation including sample collection, using these in the context of our study to obtain a clearer understanding of the feasibility of scale-up. Systematic assessment of such measures may be useful to researchers planning similar data and sample collections in other low- and middle-income populations.

While most outcomes assessed here indicated successful establishment of study operations, we identified two areas requiring improvement, which may be successfully addressed through simple modifications to study design and training. This included the slow study pace relative to the number of field teams and time taken per session of data and sample collection. This survey design-related issue was likely a result of the notable proportion of houses empty upon approach, due to outmigration or unavailability of household members at the time of recruitment. This, along with the predominantly rural setting and large sampling area, increased the travel time between houses with consenting individuals. More suitable methods of recruitment to improve study efficiency could include approaching sampled households in a separate recruitment drive to establish availability, willingness to participate, and to arrange convenient time windows for data and sample collection. We also observed lower blood sample collection success among obese participants, an issue specific to biomarker collection which may be resolved by further directed training of study phlebotomists.

The proportion of participating individuals in this study, along with differential response to participation across demographic subgroups, may suggest implications for generalisability. Although the demographic profile of this study may not be fully representative of the wider population, analyses arising from this study have the capacity to produce internally valid results regarding aetiological relationships, with wider relevance to other populations (11). Nonetheless, our observations indicate an opportunity to further improve recruitment strategies overall and across specific subgroups, in future data and sample collections.

The high burden of cardiometabolic risk factors observed in the current study population is consistent with previous findings from the SEACO HDSS (26, 27). Similar trends have been reported in other middle-income countries including those from Asia, and are thought to be a result of epidemiologic transitions occurring in these populations (28–31). These observations reinforce the need for large-scale biomarker data from such populations to comprehensively assess disease risk and associated influences across the life course. We demonstrate here that existing HDSS resources can be successfully augmented to achieve this purpose.

We present a study undertaken within a specific context, with basic infrastructure and resources already in place through the SEACO HDSS and augmented by collaborating institutions. Given our context and particular interests, we made specific choices regarding study design, including biological samples of interest, consent structure, the collection of non-fasting blood samples, and test result feedback and onward referral of participants. Researchers planning biomarker collections in other settings must consider their specific contexts and aims to inform decisions relating to suitable study design. Importantly, the measures presented here may be applicable and useful to understanding the feasibility of such biomarker collections regardless of exact study methodology.

To conclude, we show that biological sample collections to create biodata resources using existing HDSS frameworks are feasible. Using this approach, we identify a potentially high burden of cardiometabolic risk factors that requires further evaluation in this population. Building upon existing HDSS resources in this way would greatly enhance their scientific value, and contribute towards addressing the need for comprehensive biomarker data from low- and middle-income populations.

## Supplementary material

For supplementary material accompanying this paper visit https://doi.org/10.1017/gheg.2018.13. click here to view supplementary material

## References

[1] Wang H, Naghavi M, Allen C, Barber RM, Bhutta ZA, Carter A, Global, regional, and national life expectancy, all-cause mortality, and cause-specific mortality for 249 causes of death, 1980–2015: a systematic analysis for the Global Burden of Disease Study 2015. Lancet 388, 1459–544. 10.1016/S0140-6736(16)31012-1 PMC5388903 27733281

[2] Vos T, Allen C, Arora M, Barber RM, Bhutta ZA, Brown A, Global, regional, and national incidence, prevalence, and years lived with disability for 310 diseases and injuries, 1990–2015: a systematic analysis for the Global Burden of Disease Study 2015. Lancet 388, 1545–602. 10.1016/S0140-6736(16)31678-6 PMC5055577 27733282

[3] DHS. Biomarker Field Manual - Demographic and Health Survey. Maryland: ICF International/Demographic and Health Surveys; 2012.

[4] Power C, Kuh D, Morton S (2013) From developmental origins of adult disease to life course research on adult disease and aging: insights from birth cohort studies. Annu Rev Public Health 34, 7–28. 23514315 10.1146/annurev-publhealth-031912-114423

[5] Partap U, Young EH, Allotey P, Soyiri IN, Jahan N, Komahan K, (2017) HDSS Profile: The South East Asia Community Observatory Health and Demographic Surveillance System (SEACO HDSS). Int J Epidemiol 46, 1370–1 g. 29024948 10.1093/ije/dyx113 PMC5837190

[6] Allotey P, Reidpath DD, Devarajan N, Rajagobal K, Yasin S, Arunachalam D, (2014) Cohorts and community: a case study of community engagement in the establishment of a health and demographic surveillance site in Malaysia. Global Health Action 7, 10.3402/gha.v7.23176. PMC4013487 24804983

[7] Knipe DW, Jayasumana C, Siribaddana S, Priyadarshana C, Pearson M, Gunnell D, (2016) Feasibility of hair sampling to assess levels of organophosphate metabolites in rural areas of Sri Lanka. Environmental Research 147(Supplement C):207–11. 26894816 10.1016/j.envres.2016.02.011 PMC4829072

[8] Martin R, Safaee SD, Somsamouth K, Mounivong B, Sinclair R, Bansal S, (2013) Mixed Methods Pilot Study of Sharing Behaviors among Waterpipe Smokers of Rural Lao PDR: Implications for Infectious Disease Transmission. Int J Environ Res Public Health 10, 2120–32. 23708049 10.3390/ijerph10062120 PMC3717727

[9] Holloway K, Mathai E, Gray A (2011) Surveillance of community antimicrobial use in resource-constrained settings – experience from five pilot projects. Tropical Medicine & International Health 16, 152–61. 21138507 10.1111/j.1365-3156.2010.02695.x

[10] Gupta S, Ranjit A, Shrestha R, Wong EG, Robinson WC, Shrestha S, (2014) Surgical needs of Nepal: pilot study of population based survey in Pokhara, Nepal. World journal of surgery 38, 3041–6. 25189447 10.1007/s00268-014-2753-2

[11] Fry A, Littlejohns TJ, Sudlow C, Doherty N, Adamska L, Sprosen T, (2017) Comparison of Sociodemographic and Health-Related Characteristics of UK Biobank Participants With Those of the General Population. Am J Epidemiol 186, 1026–34. 28641372 10.1093/aje/kwx246 PMC5860371

[12] Day N, Oakes S, Luben R, Khaw KT, Bingham S, Welch A, (1999) EPIC-Norfolk: study design and characteristics of the cohort. European Prospective Investigation of Cancer. British journal of cancer 80 Suppl 1:95–103. 10466767

[13] Tickle-Degnen L (2013) Nuts and Bolts of Conducting Feasibility Studies. The American Journal of Occupational Therapy 67, 171–6. 23433271 10.5014/ajot.2013.006270 PMC3722658

[14] Bowen DJ, Kreuter M, Spring B, Cofta-Woerpel L, Linnan L, Weiner D, (2009) How We Design Feasibility Studies. Am J Prev Med 36, 452–7. 19362699 10.1016/j.amepre.2009.02.002 PMC2859314

[15] Kraemer HC, Mintz J, Noda A, Tinklenberg J, Yesavage JA (2006) Caution regarding the use of pilot studies to guide power calculations for study proposals. Archives of general psychiatry 63, 484–9. 16651505 10.1001/archpsyc.63.5.484

[16] Arain M, Campbell MJ, Cooper CL, Lancaster GA (2010) What is a pilot or feasibility study? A review of current practice and editorial policy. BMC Med Res Methodol 10, 67. 20637084 10.1186/1471-2288-10-67 PMC2912920

[17] Thabane L, Ma J, Chu R, Cheng J, Ismaila A, Rios LP, (2010) A tutorial on pilot studies: the what, why and how. BMC Med Res Methodol 10, 1. 20053272 10.1186/1471-2288-10-1 PMC2824145

[18] Arnold DM, Burns KE, Adhikari NK, Kho ME, Meade MO, Cook DJ (2009) The design and interpretation of pilot trials in clinical research in critical care. Crit Care Med 37(1 Suppl):S69–74. 19104228 10.1097/CCM.0b013e3181920e33

[19] Lancaster GA, Dodd S, Williamson PR (2004) Design and analysis of pilot studies: recommendations for good practice. Journal of evaluation in clinical practice 10, 307–12. 15189396 10.1111/j..2002.384.doc.x

[20] Halpern SD, Karlawish JH, Berlin JA (2002) The continuing unethical conduct of underpowered clinical trials. JAMA 288, 358–62. 12117401 10.1001/jama.288.3.358

[21] Grimes DA, Schulz KF Descriptive studies: what they can and cannot do. Lancet 359, 145–9. 11809274 10.1016/S0140-6736(02)07373-7

[22] Khowaja AR, Qureshi RN, Sawchuck D, Oladapo OT, Adetoro OO, Orenuga EA, (2016) The feasibility of community level interventions for pre-eclampsia in South Asia and Sub-Saharan Africa: a mixed-methods design. Reproductive Health 13, 56. 27357579 10.1186/s12978-016-0133-0 PMC4943500

[23] Wuest J, Merritt-Gray M, Dubé N, Hodgins MJ, Malcolm J, Majerovich JA, (2015) The Process, Outcomes, and Challenges of Feasibility Studies Conducted in Partnership With Stakeholders: A Health Intervention for Women Survivors of Intimate Partner Violence. Res Nurs Health 38, 82–96. 25594917 10.1002/nur.21636 PMC4305208

[24] Lancaster GA, Campbell MJ, Eldridge S, Farrin A, Marchant M, Muller S, (2010) Trials in primary care: statistical issues in the design, conduct and evaluation of complex interventions. Statistical methods in medical research 19, 349–77. 20442193 10.1177/0962280209359883

[25] Sy AU (2017) *Acceptability and feasibility of a community based participatory research project comparing cytology and urine HPV DNA testing for cervical cancer screening in Yap, Federated States of Micronesia*. 50, 283–8. 10.1016/j.canep.2017.07.008 PMC5739880 29120838

[26] Pell C, Allotey P, Evans N, Hardon A, Imelda JD, Soyiri I, (2016) Coming of age, becoming obese: a cross-sectional analysis of obesity among adolescents and young adults in Malaysia. BMC Pub Health 16, 1082. 27737680 10.1186/s12889-016-3746-x PMC5064972

[27] Partap U, Young E, Allotey P, Sandhu M, Reidpath D (2017) Anthropometric and cardiometabolic risk factors in parents and child obesity in Segamat, Malaysia. Int J Epidemiol (in press). 10.1093/ije/dyx114. PMC5837730 29106558

[28] Omran AR (2005) The epidemiologic transition: a theory of the epidemiology of population change. The Milbank Quarterly 83, 731–57. 16279965 10.1111/j.1468-0009.2005.00398.x PMC2690264

[29] Ghazali SM, Seman Z, Cheong KC, Hock LK, Manickam M, Kuay LK, (2015) Sociodemographic factors associated with multiple cardiovascular risk factors among Malaysian adults. BMC Pub Health 15, 68. 25636327 10.1186/s12889-015-1432-z PMC4319230

[30] Ali MK, Bhaskarapillai B, Shivashankar R, Mohan D, Fatmi ZA, Pradeepa R, (2015) Socioeconomic status and cardiovascular risk in urban South Asia: The CARRS Study. European journal of preventive cardiology. 10.1177/2047487315580891 PMC5560768 25917221

[31] Pipatvanichgul B, Hanchaiphiboolkul S, Puthkhao P, Tantirittisak T, Towanabut S (2015) Association between Socioeconomic Status and Major Risk Factors of Stroke: Thai Epidemiologic Stroke (TES) Study. Journal of the Medical Association of Thailand = Chotmaihet thangphaet 98, 739–47. 26437530

