# Supplementary material for: A biomarker feasibility study in the South East Asia Community Observatory health and demographic surveillance system
Source: Glob Health Epidemiol Genom. 2018 Aug 22;3:e14. doi: 10.1017/gheg.2018.13 (PMC6152490; doi:10.1017/gheg.2018.13)
Supplement: Supplementary file 1 [file S2054420018000131sup001.docx]

**A biomarker feasibility study in the South East Asia Community Observatory health and demographic surveillance system – Supplementary Methods**

**Study design: overview**

This feasibility study aimed to recruit approximately 200 participants aged seven years and above included in the most recent HDSS enumeration (2015-2016). Stratified random sampling was performed at the household level, and aimed to achieve comparable proportions of individuals from each of the four major ethnic groups in the area (Malay, Indian, Chinese and Orang Asli).

Community-based data and sample collection was undertaken between 11^th^ November 2016 and 16^th^ February 2017. Households that were empty upon approach three times within a three week period were listed as non-responding and not contacted further. Members of households refusing to participate were asked to provide any reasons for doing so.

Data and sample collection was undertaken in the homes of consenting individuals. Informed consent was obtained for adult participants. Informed assent with parental or guardian consent was obtained for child participants, defined in accordance with Malaysia Medical Research Ethics Committee guidelines as individuals aged 7-17 years (1). Participants consented to storage and future testing of samples, including genetic testing of blood samples, tests for environmental chemicals of hair samples, and renal function tests of urine samples, without return of results. We explored the feasibility of an all-or-none consent structure, where individuals could only participate if they consented to providing all data and samples (questionnaire data and biophysical measures, finger-prick blood and venous blood for all, and hair and urine samples for adults). This was due to the challenges partial consent (e.g. to provide only data or certain samples) presents in terms of missing data. Furthermore, we were particularly interested in blood samples, which we anticipated would most likely be refused if participants were provided options for partial consent. Nonetheless, participants were free to change their mind and withdraw from the study at any point during or after data or sample collection.

An electronic questionnaire was then administered, which recorded lifestyle risk factors and biophysical measurements including anthropometry and blood pressure. A point-of-care glycated haemoglobin (POC HbA1c) test using capillary blood (collected via finger prick) was then administered, and the results were recorded on the electronic questionnaire form. Venous blood (four tubes from a single blood draw – up to 24ml from adults and 12 ml from children; Table SM1), hair (2) and urine samples were then collected (hair and urine for adults only). There was no minimum amount for sample collection. Barcodes of biological sample containers were scanned into each participant’s questionnaire form, and sample collection status was recorded. Blood and urine samples were stored at 2-8°C, and hair samples were stored in darkness in ambient temperature in the field.

Following sample collection, participants were given their body mass index (BMI), blood pressure and POC HbA1c results, and were provided referral to local clinics if these were above cut-offs determined in consultation with local clinicians. Participants were also asked to provide feedback regarding their experience, and presented with a small token as remuneration for participating. Biological samples were transported to an accredited central research laboratory the same day. Blood samples were processed where necessary, analysed for a range of biomarkers, and aliquoted and stored for future analysis (see Table SM1). Urine samples were aliquoted and stored, along with hair samples, for later analysis. Following processing and analysis as appropriate, samples were ultimately stored at -80°C for long term storage. Two separate databases containing blood test results and the status of biological sample containers were generated.

Participants were made aware at the time of consent that they would not receive any further results from laboratory testing of biological samples. This decision was made given that we were testing the feasibility of establishing processes for biological sample handling, including collection, transportation, analysis and long-term storage.

| Table SM1. Venous blood samples collected and corresponding analysis and storage specifications. | | | | |
| --- | --- | --- | --- | --- |
| **Tube** | **Volume** | | **Analysis** | **Samples for storage** |
|  | **Adults** | **Children** |  |  |
| Plain serum | 10 ml | 5 ml | Biochemical markers | Serum |
| EDTA 1 | 6 ml | 3 ml | Unspecified (future)^1^ | Plasma |
| EDTA 2 | 6 ml | 2 ml | Genetic analysis (future)^1^ | Whole blood |
| EDTA 3 | 2 ml | 2 ml | Haematological and glycemic biomarkers | Whole blood (post analysis)^2^ |
| EDTA: ethylene diamine tetra-acetic acid. | | | | |
| ^1^Analyses were not undertaken as part of this study, to be done in future. | | | | |
| ^2^Any whole blood remaining following analysis for haematological and glycemic markers. | | | | |

A mobile application was developed to support the tracking and quality assurance of various biological samples from collection, through transportation, storage and retrieval.

Ethical approval for this study was obtained from the MUHREC (project number CF16/471 – 2016000227). Ethical approval for the receipt and analysis of linked anonymised data from this study at the University of Cambridge was obtained from the University HBREC (application number HBREC.2017.04).

**Definitions of cardiometabolic risk factors among adults and children**

Insufficient physical activity was defined as less than 150 minutes of moderate or 75 minutes of vigorous physical activity, or an equivalent combination, per week (3). The average number of fruits and vegetables consumed per day was calculated, and low fruit and vegetable consumption was defined as having less than five servings of fruit and vegetables per day (4).

Among adults, overweight was defined as BMI 25 kg/m^2^ or higher, and obesity as 30 kg/m^2^ or higher (5). Central obesity was defined as waist circumference ≥90 cm in men and ≥80 cm in women (6). High waist-to-hip ratio (WHR) was defined as WHR ≥0.9 in men and ≥0.85 in women (7). The mean of the final two of three blood pressure measurements was generated, and systolic and diastolic hypertension were defined as mean systolic and diastolic blood pressure ≥140 mmHg and ≥90 mmHg respectively (8). A composite binary measure of hypertension was defined as having measured systolic or diastolic hypertension, or history of raised blood pressure or hypertension.

For children, sex-specific BMI-for-age z-scores were calculated according to the World Health Organization (WHO) 2007 reference, and overweight and obesity were defined as BMI-for-age greater than one and two standard deviations (SD) above the mean respectively. Underweight was defined as BMI-for-age greater than two SD below the mean. Height-for-age z-scores were similarly expressed using the WHO 2007 reference, and stunting was classified as height-for-age greater than two SD below the mean (9, 10). Central obesity among children was classified according to age- and sex-specific waist circumference reference curves, derived from the National Health and Nutrition Examination Survey population, as waist circumference greater than or equal to the 90^th^ percentile (11). Hypertension was classified in accordance with National High Blood Pressure Education Program reference data and guidelines as blood pressure greater than or equal to the 95^th^ percentile for age, sex and height (12), using the EBMCalc online calculator to derive percentiles (13, 14).

For both adults and children, elevated HbA1c was defined as point-of-care HbA1c ≥6.5% (15). Anaemia was classified as haemoglobin <11.5 g/dL among children aged 5 to less than 12 years, <12.0 g/dL among individuals aged 12 to less than 15 years and non-pregnant women aged 15 years and above, <11.0 g/dL among pregnant women, and <13.0 g/dL among men aged 15 years and above (16). Elevated total cholesterol was defined as total cholesterol ≥6.21 mmol/L among adults (17), and as ≥5.18 mmol/L among children (18). Low high-density lipoprotein (HDL) cholesterol was defined as HDL cholesterol <1.04 mmol/L for both adults and children (17, 18). Elevated triglycerides (TG) was defined as serum triglycerides greater ≥2.26 mmol/L among individuals aged 20 years or above, ≥1.13 mmol/L among children aged 9 years and below, and ≥1.47 mmol/L among individuals aged 10-19 years (17, 18).

**References**

1. MREC. Guidelines Health Research Involving Minors Kuala Lumpur: Malaysia Medical Research Ethics Committee; 2011.

2. USC. Biomarker Collection Protocols - Hair Davis: University of Southern California Davis School of Gerontology [Available from: <http://gero.usc.edu/CBPH/network/resources/hair.html>.

3. WHO. Global recommendations on Physical Activity for health: World Health Organization; 2010.

4. WHO, FAO. Diet, Nutrition and the Prevention of Chronic Diseases. Geneva: World Health Organization, Food and Agriculture Organization; 2003.

5. WHO. Obesity and overweight. Geneva: World Health Organization [updated January 2015. Available from: <http://www.who.int/mediacentre/factsheets/fs311/en/>.

6. IDF. The IDF consensus on the definition of the metabolic syndrome in children and adolescents. Belgium: International Diabetes Federation; 2007.

7. WHO. Waist circumference and waist-hip ratio: Report of a WHO expert consultation, Geneva, 8-11 December 2008. 2011.

8. Whitworth JA. 2003 World Health Organization (WHO)/International Society of Hypertension (ISH) statement on management of hypertension. J Hypertens. 2003;21(11):1983-92.

9. WHO. Growth reference 5-19 years - Application tools. Geneva: World Health Organization; 2014 [Available from: <http://www.who.int/growthref/tools/en/>.

10. de Onis M, Onyango AW, Borghi E, Siyam A, Nishida C, Siekmann J. Development of a WHO growth reference for school-aged children and adolescents. Bull World Health Organ. 2007;85(9):660-7.

11. Fernández JR, Redden DT, Pietrobelli A, Allison DB. Waist circumference percentiles in nationally representative samples of African-American, European-American, and Mexican-American children and adolescents. J Pediatr. 2004;145(4):439-44.

12. NHBPEP. The fourth report on the diagnosis, evaluation, and treatment of high blood pressure in children and adolescents. Pediatrics. 2004;114(2 Suppl 4th Report):555-76.

13. Blood Pressure Percentiles for Girls (2-17 years): EBMCalc; 2017 [Available from: <http://www.hsls.pitt.edu/medcalc/BloodPressurePercentGirls.htm>.

14. Blood Pressure Percentiles for Boys (2-17 years): EBMCalc; 2017 [Available from: <http://www.hsls.pitt.edu/medcalc/BloodPressurePercentBoys.htm>.

15. IDF. Global Guideline for Type 2 Diabetes. Brussels International Diabetes Federation; 2012.

16. Benoist Bd, McLean E, Egll I, Cogswell M. Worldwide prevalence of anaemia 1993-2005: WHO global database on anaemia. Worldwide prevalence of anaemia 1993-2005: WHO global database on anaemia. 2008.

17. Third Report of the National Cholesterol Education Program (NCEP) Expert Panel on Detection, Evaluation, and Treatment of High Blood Cholesterol in Adults (Adult Treatment Panel III) Final Report. Circulation. 2002;106(25):3143.

18. NHLBI. Expert Panel on Integrated Guidelines for Cardiovascular Health and Risk Reduction in Children and Adolescents. Bethesda: National Heart Lung and Blood Institute; National Institutes of Health; 2012.
